# Vitamin D, Albumin, and D-Dimer as Significant Prognostic Markers in Early Hospitalization in Patients with COVID-19

**DOI:** 10.3390/jcm12082825

**Published:** 2023-04-12

**Authors:** Biljana Popovska Jovičić, Ivana Raković, Jagoda Gavrilović, Sofija Sekulić Marković, Sara Petrović, Vladan Marković, Aleksandar Pavković, Predrag Čanović, Ružica Radojević Marjanović, Violeta Irić-Čupić, Lidija Popović Dragonjić, Miloš Z. Milosavljević

**Affiliations:** 1 Department of Infectious Diseases, Faculty of Medical Sciences, University of Kragujevac, 34000 Kragujevac, Serbia; 2 Clinic for Infectious Diseases, University Clinical Center Kragujevac, Zmaj Jovina 30, 34000 Kragujevac, Serbia; 3 Department of Radiology, Faculty of Medical Sciences, University of Kragujevac, 34000 Kragujevac, Serbia; 4 Department of Radiological Diagnostics, University Clinical Center Kragujevac, 34000 Kragujevac, Serbia; 5 Department of Internal Medicine, Faculty of Medical Sciences, University of Kragujevac, 34000 Kragujevac, Serbia; 6 Clinic for Cardiology, University Clinical Center Kragujevac, 34000 Kragujevac, Serbia; 7 University of Niš, Faculty of Medicine in Nis, Cathedra for Infectious Diseases and Epidemiology, Blvd. Dr Zorana Djindjica 81, 18000 Niš, Serbia; 8 Clinic for Infectology, University Clinical Center Niš, 18000 Niš, Serbia; 9 Department of Pathology, University Clinical Center Kragujevac, 34000 Kragujevac, Serbia

**Keywords:** COVID-19, vitamin D, albumin, D-dimer, severity of illness, SARS-CoV-2, lethal outcome

## Abstract

SARS-CoV-2 continues to pose a major challenge to scientists and clinicians. We examined the significance of the serum concentrations of vitamin D, albumin, and D-dimer for the severity of the clinical picture and mortality in COVID-19. Materials and methods: A total of 288 patients treated for COVID-19 infection participated in the research. The patients were treated in the period from May 2020 to January 2021. All patients were divided based on the need for oxygen therapy (Sat > 94%) into patients with mild or severe clinical pictures. The biochemical and radiographic parameters of the patients were analyzed. Appropriate statistical methods were used in the statistical analysis. Results: In patients with COVID-19 with confirmed severe clinical pictures, lower values of serum albumin (*p* < 0.0005) and vitamin D (*p* = 0.004) were recorded, as opposed to elevated values of D-dimer (*p* < 0.0005). Accordingly, the patients with fatal disease outcomes had lower levels of albumin (*p* < 0.0005) and vitamin D (*p* = 0.002), while their D-dimer (*p* < 0.0005) levels were elevated. An increase in the radiographic score, as a parameter for assessing the severity of the clinical picture, was accompanied by a decrease in serum albumin (*p* < 0.0005) and a simultaneous increase in D-dimer (*p* < 0.0005), without a change in the vitamin D concentration (*p* = 0.261). We also demonstrated the interrelations of the serum levels of vitamin D, albumin, and D-dimer in patients with COVID-19 as well as their significance as predictors of the outcome of the disease. Conclusion: The significance of the predictive parameters in our study indicates the existence of an important combined role of vitamin D, albumin, and D-dimer in the early diagnosis of the most severe patients suffering from COVID-19. Reduced values of vitamin D and albumin, in combination with elevated values of D-dimer, can be timely indicators of the development of a severe clinical picture and death due to COVID-19.

## 1. Introduction

The causative agent of COVID-19 is a form of the SARS coronavirus (SARS-CoV) known as severe acute respiratory syndrome coronavirus 2 (SARS-CoV-2) [1]. Coronaviruses (COVs) are a group of extremely diverse, enveloped, positive-sense, and single-stranded RNA viruses [2]. Most of them cause common cold symptoms, but there are exceptions such as SARS-CoV and Middle East respiratory syndrome coronavirus (MERS-CoV), which are far more pathogenic than the others and cause fatal illness [3]. SARS-CoV-2 is the seventh new COV that can infect humans. It belongs to the β-COV group and is extremely infectious, causing human-to-human transmission. The spike protein (S) is important for coronavirus transmission, as it mediates receptor binding and the membrane fusion of the virus to the host angiotensin-converting enzyme 2 (ACE2), a cell receptor for SARS-CoV [4].

Various organs such as the lungs, heart, arteries, kidneys, intestine, etc., possess ACE2 attached to their cell membranes [5]. The spectrum of the clinical manifestations of COVID-19 ranges from asymptomatic to severe respiratory failure, with symptoms including fever, exhaustion, the loss of the senses of smell and taste, croup, runny nose, cough, shortness of breath, etc. [6]. Since the beginning of the COVID-19 pandemic, research efforts have been aimed at identifying reliable risk factors and prognostic factors for severe forms of the disease. The most common of these include old age, comorbidities (e.g., chronic heart, kidney, and lung diseases; diabetes; hypertension; and obesity), lymphocytopenia, and elevated levels of C-reactive protein (CRP) and D-dimer [7].

Vitamin D is one of the factors that has been evaluated for its influence on COVID-19. Comprehensive review papers have indicated possible protective effects of vitamin D in numerous viral infections (HIV-1, HSV1/2, hepatitis B and C, rotavirus, influenza, respiratory syncytial virus, etc.). They also found that it reduces the risk of acute respiratory infections [8,9]. The evidence for the relation between serum levels of vitamin D and the severity and disease outcomes in patients with COVID-19 is conflicting. Some studies observed no significant differences in 25-hydroxyvitamin D (25(OH)D) serum levels among critically ill adult persons who recovered and those who died [10], while other studies connected the vitamin D, body mass, and age of patients to the prognosis for developing severe COVID-19 disease [11].

The antiviral effects of vitamin D had been described long before the COVID-19 pandemic. Vitamin D exerts favorable effects on the T-cell immune response by reducing the Th1 immune response and inducing the anti-inflammatory Th2 immune response [12]. The results of recent research indicate that the active forms of vitamin D have anti-inflammatory effects in COVID-19 by inhibiting interleukin (IL)-1, IL-6, IL-17, tumor necrosis factor-α, and interferon-γ [13]. According to other authors, low levels of vitamin D are associated with increased levels of inflammatory cytokines and more severe forms of the disease [14]. Some authors suggest that vitamin D could reduce the number of ACE-2 receptors and thus have a protective effect in COVID-19 [15].

The results of various studies have shown that hypoalbuminemia is common in patients with COVID-19 and that it is significantly associated with disease severity and a poor prognosis [16]. A relationship between the albumin levels at admission and the risk of severe infection and death has been established [17]. It is assumed that hypoalbuminemia in COVID-19 develops not only due to the damage to the hepatocellular system but also as a result of systemic inflammation and increased capillary permeability, which causes albumin to disappear into the interstitium [18]. Hypoalbuminemia represents an index of systemic inflammatory response severity, and it has been shown that it has a predictive significance in COVID-19 [19].

D-dimer is a soluble product of fibrin degradation that occurs during the disintegration of the thrombus by the actions of the fibrinolytic system. Numerous studies have shown that D-dimer serves as a significant indicator of activated coagulation and fibrinolysis. Therefore, it is used for the diagnosis of venous thromboembolism (VTE), to monitor disseminated intravascular coagulation, and for identifying patients at high risk of VTE [20]. An elevated D-dimer level has been recognized since the beginning of the pandemic as a significant predictor of the severity and mortality of COVID-19 [21]. In the early stages of COVID-19, concentrations of D-dimer that are increased by 3 to 4 times, in combination with fibrinogen, are associated with a poor prognosis [22].

We are not aware of any published studies aiming to evaluate vitamin D, albumin, and D-dimer as prognostic factors of COVID-19 severity and outcomes. Considering all of the above, there is a need for research to assess the relationship between vitamin D, albumin, and D-dimer and the severity and outcomes of COVID-19.

## 2. Materials and Methods

**Figure 1:**
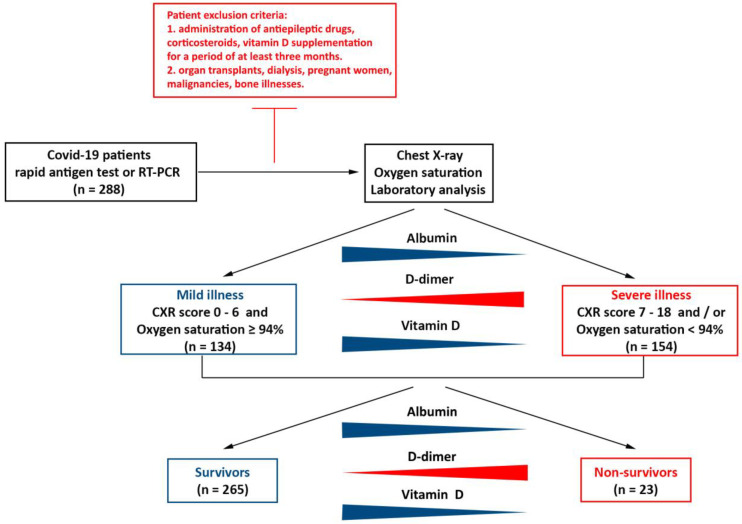
Graphic representation of the entire workflow of the study. (chest X-ray (CXR), real-time reverse transcription (RT-PCR)).

The study was designed as an observational, cross-sectional study. It included 288 patients with COVID-19 infection confirmed by either a rapid antigen test or by real-time reverse transcription (RT-PCR) analysis (Figure 1). The patients were treated in the period from May 2020 to January 2021.

The patients had not previously been receiving antiepileptics, corticosteroids, or vitamin D supplementation for a period of at least three months prior to the study’s onset. We excluded patients with organ transplants, those receiving dialysis, pregnant women, and those with malignancies and bone illnesses (osteoporosis, Padget’s disease, and osteomalacia).

All patients were divided into two groups according to the need for oxygen therapy and chest X-ray findings:

A total of 134 patients had mild illness, which did not require oxygen therapy (oxygen saturation level ≥ 94%) and had chest X-ray (CXR) findings that were normal or had a marked infiltrative pattern.

A total of 154 patients had severe illness, which required oxygen therapy (oxygen saturation level < 94%) and/or had CXR findings with focal or multifocal lung tissue consolidation.

Depending on the clinical severity, the patients were treated with different modes of oxygen therapy: O_2_ delivered via a nasal cannula or a mask, high-flow ventilation, noninvasive ventilation, and mechanical ventilation.

The CXR findings were analyzed using the method published by Borghesi et al. in 2020. According to the methodology of that paper, the lung images were divided by two lines into six zones. Then, each zone was scored based on changes in the lung tissue: 0—no changes in the lung tissue, 1—interstitial infiltrative pattern, 2—alveolar infiltrates, and 3—interstitial and alveolar infiltrates. By adding the individual scores of each zone, we obtained the total score, ranging from 0 to 18 (CXR) [23]. According to this score, the CXR findings were categorized as normal (CXR 0), with diffuse marked interstitial infiltrative patterns (CXR 1–6), focal consolidation of lung tissue (CXR 7–12), and multifocal consolidation (CXR 13–18).

We analyzed the differences in D-dimer, serum albumin, and vitamin D levels in all patients, as well as their relation with illness severity and outcome. Blood samples of COVID-19-positive patients were obtained by phlebotomy, immediately upon admission, for complete blood count (CBC), coagulation tests (INR, PV, and fibrinogen), and biochemical analysis (C-reactive protein (CRP), procalcitonin (PCT), glycemia, aspartate aminotransferase (AST), alanine aminotransferase (ALT), creatine kinase MB (CK-MB), lactate dehydrogenase (LDH), N-terminal (NT)-pro hormone BNP (NT-proBNP), troponin, ferritin, and potassium (K)). Each laboratory analysis was performed in the central laboratory of the University Clinical Center Kragujevac using standard methods on a Beckman Coulter AU 400 Unicel DXC 800 Synchron Clinical System.

A body fat assessment based on body mass and height was performed by calculating the body mass index (BMI) [24].

BMI = (body mass (kg))/(height (m))^2^

This study was performed at the University Clinical Center Kragujevac (COVID-19 Center). The Ethics Committee of the University Clinical Center Kragujevac issued the approval (approval number: 01/20-493). All participants gave their written consent. All research procedures were carried out in accordance with the Declaration of Helsinki and the Principles of Good Clinical Practice.

## 3. Statistical Analysis

Statistical analysis was performed using the SPSS software package, version 26 (SPSS Inc., Chicago, IL, USA). To compare the mean values, depending on the number of groups being examined, we applied the Mann–Whitney U test or the Kruskal–Wallis test. The relationships of the two continuous variables were analyzed using the Spearman correlation coefficient. For the analysis of 2 × 2 contingency tables, the Fisher’s exact test was used. The influence of predictor variables on the clinical severity and disease outcome was analyzed using a binary logistic regression. The reliability of the laboratory parameters (D-dimer, vitamin D, and albumin) as predictors of clinical severity and outcome, with the simultaneous determination of the cut-off values, sensitivity, specificity, and AUROC (area under the ROC curve), was assessed using an ROC curve analysis. Statistical significance was confirmed at *p* < 0.05. The results of comparison between groups were presented as median (1Q, 3Q).

## 4. Results

### 4.1. Decreased Levels of Vitamin D and Albumin, in Combination with Elevated Levels of D-Dimer, Indicate a More Severe Clinical Picture and a Fatal Outcome for Patients with COVID-19

In 288 patients with COVID-19, we analyzed the differences in the values of D-dimer, albumin, and vitamin D in relation to the severity of the clinical picture and the outcome of the disease. The clinical characteristics of the studied patients are shown in Table 1.

**Figure 2:**
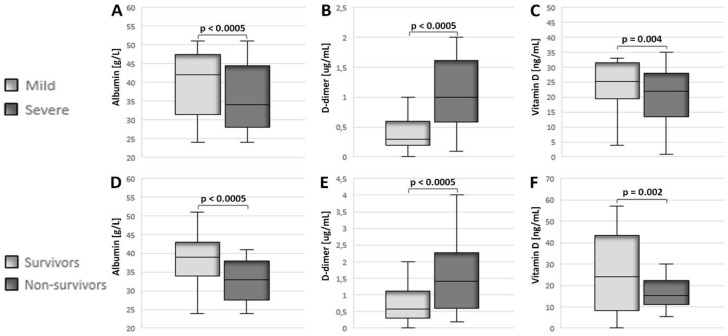
Serum values of albumin, D-dimer, and vitamin D in patients with COVID-19 in relation to the severity of the clinical picture and the outcome of the disease. The patients were divided into groups with mild (n = 134) and severe (n = 154) clinical pictures as well as groups with favorable (n = 265) and fatal (n = 23) disease outcomes. The serum values of the examined biochemical parameters were obtained from blood samples immediately after admission to the hospital for treatment. (**A**) Differences in serum albumin (Mdn = 42.0 g/L (39.0, 44.0), n = 134 vs. Mdn = 34.0 g/L (32.0, 38.0), n = 154; *p* < 0.0005); (**B**) D-dimer (Mdn = 0.3 ug/mL (0.2, 0.6), n = 134 vs. Mdn = 1 ug/mL (0.6, 1.6), n = 154; *p* < 0.0005); and (**C**) vitamin D (Mdn = 25.1 ng/mL (19.6, 31.4), n = 134 vs. Mdn = 22.0 ng/mL (13.5, 27.9), n = 154; *p* = 0.004) values in patients with mild and severe clinical pictures. (**D**) Differences in the values of serum albumin (Mdn = 39.0 g/L (34.0, 43.0), n = 265 vs. Mdn = 33.0 g/L (31.0, 35.0), n = 23; *p* < 0.0005); (**E**) D-dimer (Mdn = 0.6 ug/mL (0.3, 1.1), n = 265 vs. Mdn = 1.4 ug/mL (0.6, 2.3), n = 23; *p* < 0.0005); and (**F**) vitamin D (Mdn = 24.1 ng/mL (16.4, 29.5), n = 265 vs. Mdn = 15.1 ng/mL (11.1, 22.2), n = 23; *p* = 0.002) in patients with favorable and fatal disease outcomes. Statistical significance was examined using the Mann–Whitney U test. Results were presented as median (1Q, 3Q). Values of *p* < 0.05 were considered statistically significant.

In patients with COVID-19 with a confirmed severe clinical picture, lower values of serum albumin and vitamin D were recorded (Figure 2A,C), as opposed to an elevated value of D-dimer (Figure 2B). In accordance with this, the patients with fatal disease outcomes had lower levels of albumin and vitamin D (Figure 2D,F), while their D-dimer levels were elevated (Figure 2E).

**Figure 3:**
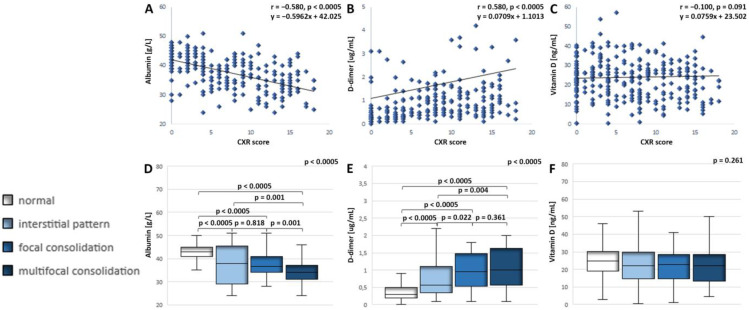
Serum albumin and D-dimer levels are associated with radiographic lung changes in patients with COVID-19. The serum values of the examined biochemical parameters were obtained from blood samples immediately after admission to the hospital for treatment. Chest X-ray images were divided by two lines into six zones. Each zone was scored based on the changes in the lung tissue. Then, the CXR score was calculated as the sum of the individual scores of each zone (range: 0 to 18). According to the CXR score, the findings were categorized as normal, diffuse marked interstitial patterns, focal consolidation of lung tissue, or multifocal consolidation (n = 288). (**A**) Correlations of serum albumin; (**B**) D-dimer; and (**C**) vitamin D values with the lung changes expressed by the CXR score. Based on the CXR score, the lung findings were classified as normal, accentuated interstitium, and focal or multiple consolidation. (**D**) Differences in the values of serum albumin (Mdn = 43.0 g/L (41.0, 45.0), n = 96 vs. Mdn = 38 g/L (34.0, 40.0), n = 44 vs. Mdn = 36.5 g/L (34.0, 41.0), n = 56 vs. Mdn = 34.0 g/L (31.0, 37.0), n = 91; *p* < 0.0005); (**E**) D-dimer (Mdn = 0.3 ug/mL (0.2, 0.5), n = 96 vs. Mdn = 0.6 ug/mL (0.3, 1.1), n = 44 vs. Mdn = 1.1 ug/mL (0.5, 1.5), n = 56 vs. Mdn = 1.0 ug/mL (0.6, 1.7), n = 91; *p* < 0.0005); and (**F**) vitamin D (Mdn = 24.8 ng/mL (19.2, 30.2), n = 96 vs. Mdn = 22.2 ng/mL (14.5, 30.0), n = 44 vs. Mdn = 22.8 ng/mL (14.6, 28.6), n = 56 vs. Mdn = 22.2 ng/mL (28.4, 13.4), n = 91; *p* = 0.261), depending on the severity of the CXR changes in the lungs. Statistical significance was examined using the Spearman correlation coefficient, the Kruskal–Wallis H test, and/or the Mann–Whitney U test. Results were presented as median (1Q, 3Q). Values of *p* < 0.05 were considered statistically significant.

An increase in the radiographic score, as a parameter for assessing the severity of the clinical picture, was accompanied by a decrease in serum albumin and a simultaneous increase in D-dimer (Figure 3A,B), without a change in the vitamin D concentration (Figure 3C). This result was confirmed by analyzing the concentrations of the examined biochemical parameters, depending on the severity of the radiographic changes, which, as previously described, were determined based on the CXR score (Figure 3D–F).

### 4.2. Serum Concentrations of Vitamin D, D-Dimer, and Albumin Are Significant Prognostic Factors for the Severity of the Clinical Picture and the Outcome of the Disease

**Figure 4:**
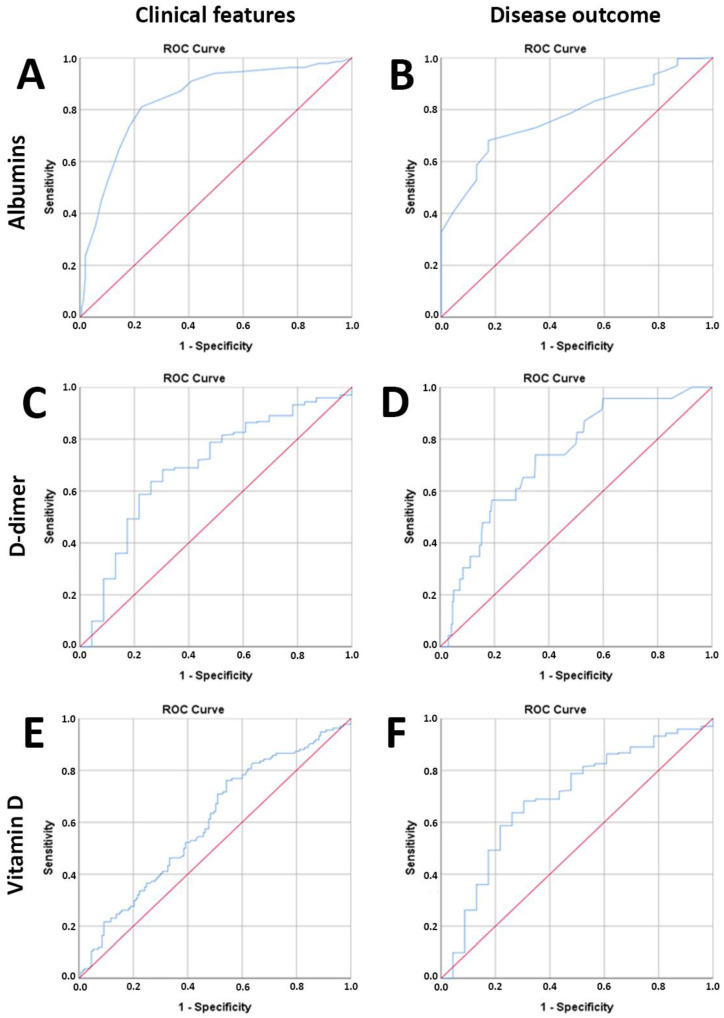
ROC analysis of albumin, D-dimer, and vitamin D as predictor parameters for evaluating the severity of the clinical picture and the outcome of the disease. The patients were divided into groups with mild (n = 134) and severe (n = 154) clinical pictures as well as groups with favorable (n = 265) and fatal (n = 23) disease outcomes. The serum values of the examined biochemical parameters were obtained from blood samples immediately after admission to the hospital for treatment. (**A**) Serum albumin values indicate the development of a severe clinical picture and (**B**) a lethal outcome. (**C**) Serum D-dimer values indicate the development of a severe clinical picture and (**D**) a fatal outcome. (**E**) Values of serum vitamin D indicate the development of a severe clinical picture and (**F**) a lethal outcome. Statistical significance was determined using the ROC curve. Values of *p* < 0.05 were considered statistically significant.

Serum albumin concentrations lower than the obtained cut-off value of 38.5 g/L indicate a severe clinical picture in patients with COVID-19 (AUROC: 0.838, sensitivity: 81.1%, specificity: 77.3%, *p* < 0.0005), while values less than 34.5 g/L may indicate a risk of a lethal disease outcome (AUROC: 0.776, sensitivity: 73.0%, specificity: 65.2%, *p* < 0.0005) (Figure 4A,B).

Furthermore, a serum concentration of vitamin D lower than 23.69 ng/mL is characteristic of patients with a severe clinical picture (AUROC: 0.599, sensitivity: 54.5%, specificity: 54.2%, *p* = 0.004), while concentrations lower than 18.83 ng/mL indicate a potentially fatal outcome for patients (AUROC: 0.692, sensitivity: 68.2%, specificity: 65.2%, *p* = 0.002) (Figure 4E,F).

Contrary to the reduced values of albumin and vitamin D, D-dimer values higher than the obtained cut-off value of 0.50 ug/mL are characteristic of COVID-19 patients with severe clinical pictures (AUROC: 0.808, sensitivity: 79.7%, specificity: 72.0%, *p* < 0.0005), while values above 0.82 ug/mL may indicate a risk of a lethal disease outcome (AUROC: 0.733, sensitivity: 73.0%, specificity: 64.9%, *p* < 0.0005) (Figure 4C,D).

### 4.3. Interrelation of Serum Levels of Vitamin D, Albumin, and D-Dimer in Patients with COVID-19

**Figure 5:**
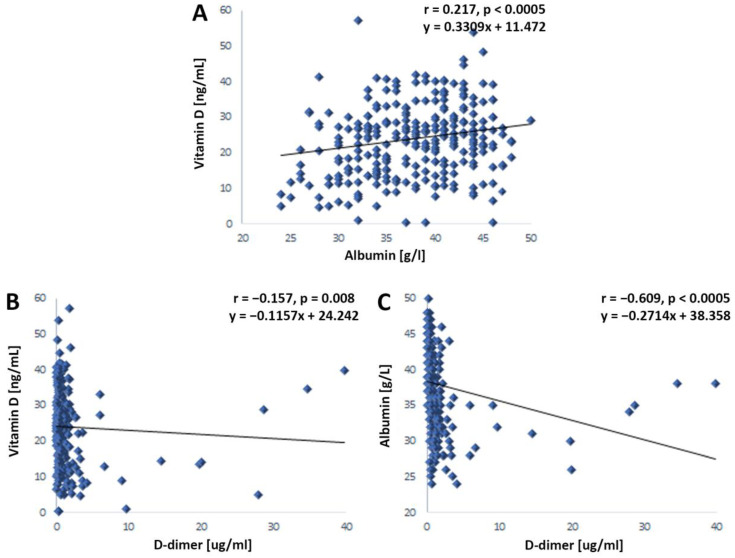
Intercorrelations of serum albumin, D-dimer, and vitamin D in patients with COVID-19. The serum values of the examined biochemical parameters were obtained from blood samples immediately after admission to the hospital for treatment (n = 288). (**A**) Correlation of albumin and vitamin D in patients with COVID-19. (**B**) Correlation of D-dimer and vitamin D in patients with COVID-19. (**C**) Correlation of D-dimer and albumin in patients with COVID-19. Statistical significance was examined using the Spearman correlation coefficient. Values of *p* < 0.05 were considered statistically significant.

Increased levels of vitamin D were accompanied by higher levels of albumin in patients with COVID-19 (Figure 5A). On the contrary, elevated levels of vitamin D were simultaneously accompanied by decreased levels of D-dimer (Figure 5B). Accordingly, increased serum albumin levels were accompanied by decreased D-dimer levels (Figure 5C).

Furthermore, we analyzed the correlations between the investigated laboratory parameters in the groups of patients with mild and severe clinical pictures. In patients with a mild clinical picture, reduced albumin values were only associated with elevated D-dimer values (Table 2). However, in patients with a severe clinical picture, we determined the same interrelationships of the investigated laboratory parameters as in the entire sample (Table 2).

### 4.4. Associations of Vitamin D, Albumin, and D-Dimer with Other Laboratory Parameters in Patients with COVID-19

The associations of serum albumin, D-dimer, and vitamin D with other laboratory parameters indicating the severity of the disease are shown in Table 3.

### 4.5. The Influence of the Examined Set of Predictors (Albumin, D-Dimer, and Vitamin D) on the Development of a Severe Clinical Picture and a Fatal Disease Outcome in Patients with COVID-19

A binary logistic regression was performed to assess the influences of multiple factors on the probability that subjects will develop a severe clinical picture of COVID-19. The model contained three independent variables (the serum values of albumin, D-dimer, and vitamin D) and, together with all predictors, was statistically significant (χ^2^ (df 3, n = 283) = 104.084, *p* < 0.0005), which indicates that the model distinguishes patients who have developed a severe clinical picture and those who have not. The model explains between 30.8% (Cox and Snell R Square) and 41.1% (Nagelkerke R Square) of the variance and correctly classifies 80.2% of cases. As shown in Table 4, only the albumin value made a unique contribution to the model, with an odds ratio of 0.759. By correcting the odds ratio, we obtained (0.759–1) × 100 = −24.1%. The obtained result indicates that an increase in the value of serum albumin by 1 g/L reduces the probability of developing a severe form of COVID-19 by 24.1%, provided that the values of all other parameters in the model are equal.

An identical model was used to assess the influences of multiple factors on the probability that subjects will develop a fatal disease outcome. This model was also statistically significant (χ^2^ (df 3, n = 283) = 22.763, *p* < 0.0005), which indicates that the model distinguishes between patients with fatal outcomes and patients with favorable outcomes. The model explains between 7.7% (Cox and Snell R Square) and 17.9% (Nagelkerke R Square) of the variance and correctly classifies 92.2% of cases. In accordance with the previous statement, only the albumin value made a unique contribution to the model (Table 5), with an odds ratio of 0.823. By correcting the odds ratio, we obtained (0.823–1) × 100 = −17.7%. The obtained result shows that an increase in the value of serum albumin by 1 g/L reduces the probability of a fatal disease outcome by 17.7%, provided that the values of all other parameters in the model are equal.

## 5. Discussion

Our results show that decreased levels of vitamin D and albumin, in combination with elevated levels of D-dimer, indicate a more severe clinical picture and a fatal outcome due to COVID-19.

Previous research has already shown the importance of vitamin D in relation to COVID-19, which is in line with our research results indicating a correlation between the level of vitamin D and the severity of the clinical picture.

Vitamin D has an immunomodulatory role in innate and acquired immunity as well as in the regulation of cytokine signaling in COVID-19 [25,26].

The anti-inflammatory role of vitamin D is also reflected in the regulation of the ACE2 levels in the lungs, which can reduce the development of a cytokine storm in severe COVID-19 and the occurrence of multiorgan dysfunction [27].

It is thought that a severe SARS-CoV2 infection can reduce the concentration of vitamin D in the early stages of infection due to the downregulation of CYP2R1, one of the six cytochromes that catalyze both forms of vitamin D (D2 and D3) [28]. There is also an assumption that during an infection there is a decrease in the level of the main vitamin D transport protein, the vitamin D binding protein (VDBP), because it binds actin and other protein complexes during the infection, causing the low levels of total vitamin D [29].

Moreover, the factors that could have contributed to low vitamin D values in our sample are common factors for which lower vitamin D values are expected, such as older age, obesity, and some comorbidities, such as diabetes. Although there is a decreasing trend with age, no significant association was found in our sample. Moreover, no significant association of vitamin D levels with BMI values was observed in our results. Regarding comorbidity, significantly lower values of vitamin D were recorded in patients with diabetes, which is in accordance with the results of other authors [30,31]. It is of particular importance to note the fact that diabetes patients predominantly (86.4%) belonged to the group of severe patients. Patients with diabetes have a weakened immune response to infection, including T-cell activation and macrophage activation, while poor glycemic control negatively affects the immune response to a viral infection as well as potential bacterial infections [30]. This is a possible explanation for their predisposition for the development of a severe clinical picture and a fatal outcome during COVID-19.

In addition to reduced vitamin D values, our results also showed that some biochemical analyses, such as serum albumin values, can have predictive and prognostic significance for the clinical picture and disease outcome.

It is believed that the decrease in the concentration of albumin in the plasma affects the concentration of vitamin D in the plasma as well as its bioavailability [32]. It should be noted that 85–90% of the total circulating vitamin D (25 (OH)D) is bound to VDBP, while about 15% is bound to albumins and less than 1% is in its free form. The 25 (OH)D fraction that is bound to VDBP is biologically inactive, while the other two fractions are biologically active, including the fraction bound to albumins and the free fraction [33]. Recent studies have shown that the albumin-bound fraction and the free fraction are much more useful markers for outcomes in more severe clinical conditions compared to the total 25 (OH)D. The bioavailable fraction bound to albumin dissociates rapidly, so a decrease in plasma albumin is thought to affect plasma vitamin D concentrations [34].

Our results showed that D-dimer values were elevated in the group of seriously ill patients and those with fatal outcomes.

In patients with cytokine storms, elevated values of D-dimer were recorded in COVID-19, which implies elevated markers of pro-inflammatory cytokines that are inadequately controlled by anti-inflammatory factors, which triggers the coagulation cascade [35].

Research by other authors has shown that elevated coagulation parameters, such as D-dimer values, indicate hypercoagulability and a worse disease prognosis [36]. It is known that increased inflammatory activity activates the coagulation system and that the coagulation system further activates inflammation. The merging of the two systems is reflected in the formation of a microvascular microthrombus, which often leads to organ dysfunction in critically ill patients with COVID-19 [37].

Many studies have shown that older patients have higher D-dimer values, most likely due to the presence of comorbidities, which was also shown in our study [22].

Elderly people with comorbidities, such as hypertension, diabetes mellitus, and cardiovascular comorbidities, as well as COVID-19 have risk factors for the development of a severe clinical picture and have a predisposition for the occurrence of thrombosis [38,39].

Given that almost all cells of the immune system express the vitamin D receptor and that it mediates several molecular mechanisms, its immunomodulatory role is also known [40]. Considering its possible anti-inflammatory role, it is considered that vitamin D can affect some processes during the coagulation cascade, so the reduced vitamin D values in our results are negatively correlated with D-dimer and indicate a severe clinical picture and death [41].

Our results showed the existence of an inverse relationship between albumin and D-dimer levels, which indicates the existence of an inextricable link between inflammation and thrombosis, which is clinically manifested by a severe clinical picture and a possible fatal outcome in COVID-19.

Our results showed that decreased albumin values were accompanied by elevated D-dimer values in patients with a severe clinical picture and a fatal outcome.

There are different hypotheses about the inverse relationship between hypoalbuminemia and high D-dimer values. In severe COVID-19, hypoalbuminemia occurs as a result of a systemic inflammatory response, i.e., SIRS [42]. Decreased albumin synthesis may be a consequence of the release of inflammatory mediators, primarily IL-1, IL-6, and TNF-α [43]. In addition, due to increased permeability of capillaries within SIRS, a loss of albumin occurs in the interstitium. At the same time, elevated D-dimer values correlate with inflammation markers such as CRP and IL-6, as in severe COVID-19 [44].

During a cytokine storm in COVID-19, in addition to elevated and uncontrolled pro-inflammatory markers, elevated D-dimer values were also recorded as a triggered coagulation cascade [45].

Moreover, associated hypoalbuminemia is a factor that favors hypercoagulability. Previous studies showed that its anticoagulant and antithrombotic properties are reflected in the inhibition of fibrin polymerase, antithrombin III, modulating factors V and VIII, and fibrinogen [46].

Various groups of authors have shown that the presence of thromboinflammation in SARS-CoV2 affects the development of hypercoagulability, which is mediated by cytokine storms and hypoxemia and leads to the development of thromboembolic complications [47,48].

Serum concentrations of vitamin D, D-dimer, and albumin are significant prognostic factors for the severity of clinical pictures and the outcome of the disease. In order to obtain more precise parameters, the albumin values were first analyzed, which showed that values lower than 38.5 indicate a severe clinical picture, while values lower than 34.5 may indicate the risk of a fatal outcome. Even lower cut-off values for lethal outcomes were obtained by Italian authors, who showed that values lower than 32 were associated with respiratory insufficiency, fatal outcomes, and long hospitalizations [49,50].

Our results showed that in patients with a severe clinical picture, values lower than 23.69 were recorded, while concentrations lower than 18.83 indicated potentially lethal outcomes for the patients. Other authors who examined vitamin D values in 551 patients showed that values lower than 12 ng/mL were associated with death, considering that vitamin D may contribute to the pro-inflammatory and prothrombin state [51].

On the other hand, D-dimer values above 0.815 may indicate a risk of a fatal outcome, suggesting lower values compared to a study published in 2021 on 182 patients whose results showed a cut-off of 1.55 μg/mL as a predictor of mortality for patients with COVID-19 [22].

The biggest limitation of this study is that it was conducted at one center and that the data were collected retrospectively. Furthermore, during the sampling period, there were several changes to the national guidelines for the treatment of COVID-19.

For all patients, blood tests were taken within the first 24 h of admission to the hospital for treatment, which certainly influenced the cut-off results that we present in the research. We assume that if we sampled the values at two or three time points, this would affect the cut-off result because, over time, some values of the laboratory parameters would change, such as the increase in the D-dimer value. Nevertheless, our results are significant precisely because they can predict the clinical course of the disease at the beginning of hospitalization.

## 6. Conclusions

The significance of predictive parameters in our study indicates the existence of an important combined role of vitamin D, albumin, and D-dimer in the early diagnosis of the most severe patients suffering from COVID-19. Reduced values of vitamin D and albumin and elevated values of D-dimer can quickly indicate the development of a severe clinical picture and death due to COVID-19. Moreover, our study suggests that the values of the examined parameters could be revised in order to better predict the most difficult patients.

## Figures and Tables

**Table 1:** Clinical and demographic characteristics of patients with COVID-19. The serum values of the examined biochemical parameters were obtained from blood samples immediately after admission to the hospital for treatment, while other data were obtained from the medical history (n = 288).

Variables		Clinical Features			Disease Outcome		
		Mild	Severe	*p* Value	Survivors	Non-Survivors	*p* Value
Patients		134 (46.5%)	154 (53.5%)	NA	265 (92.0%)	23 (8.0%)	NA
Gender	Male	77 (40.3%)	114 (59.7%)	0.004 *	175 (91.6%)	16 (8.4%)	0.821 *
	Female	57 (58.8%)	40 (41.2%)		90 (92.8%)	7 (7.2%)	
Age Mdn (1Q, 3Q)		45.5 (37.8, 59.3)	65.5 (55.0, 71.0)	<0.0005 **	57 (42.0, 67.0)	70 (67.0, 77.0)	<0.0005 **
BMI Mdn (1Q, 3Q)		24.8 (22.1, 26.8)	27.8 (26.0, 30.8)	<0.0005 **	26.3 (24.0, 29.1)	27.8 (25.8, 29.8)	0.076 **
Diabetes mellitus	No	124 (69.7%)	103 (30.3%)	<0.0005 *	214 (94.3%)	13 (5.7%)	0.012 *
	Yes	8 (26.0%)	51 (74.0%)		49 (83.1%)	10 (16.9%)	
Arterial hypertension	No	105 (62.9%)	62 (37.1%)	<0.0005 *	160 (95.8%)	7 (4.2%)	0.007 *
	Yes	27 (22.7%)	92 (77.3%)		103 (86.6%)	16 (13.4%)	
Obstructive lung disease	No	125 (46.5%)	144 (53.5%)	0.777 *	247 (91.8%)	22 (8.2%)	NA
	Yes	5 (38.5%)	8 (61.5%)		12 (92.3%)	1 (7.7%)	

* Fisher’s exact test. ** Mann–Whitney U test. Results were presented as median (1Q, 3Q). Values of *p* < 0.05 were considered statistically significant.

**Table 2:** Intercorrelations of examined biochemical parameters within groups of patients with mild and severe clinical pictures. The serum values of the examined biochemical parameters were obtained from blood samples immediately after admission to the hospital for treatment. Patients were divided into groups with mild (n = 134) and severe (n = 154) clinical pictures. Statistical significance was examined using the Spearman correlation coefficient. Values of *p* < 0.05 were considered statistically significant.

Clinical Features	Variables	Albumin (g/L)			D-dimer (ug/mL)			Vitamin D (ng/mL)		
		Spearman’s Rho	*p* Value	N	Spearman’s Rho	*p* Value	N	Spearman’s Rho	*p* Value	N
Mild	Albumin (g/L)	1.000	.	132	−0.512	<0.0005	131	−0.022	0.799	132
	D-dimer (ug/mL)	−0.512	<0.0005	131	1.000	.	132	0.054	0.540	132
	Vitamin D (ng/mL)	−0.022	0.799	132	0.054	0.540	132	1.000	.	134
Severe	Albumin (g/L)	1.000	.	154	−0.368	<0.0005	153	0.288	<0.0005	153
	D-dimer (ug/mL)	−0.368	<0.0005	153	1.000	.	153	−0.239	0.003	152
	Vitamin D (ng/mL)	0.288	<0.0005	153	−0.239	0.003	152	1.000	.	153

Spearman’s correlation coefficient. Values of *p* < 0.05 were considered statistically significant.

**Table 3:** Correlations of serum albumin, D-dimer, and vitamin D with other biochemical parameters in patients with COVID-19. The serum values of the biochemical parameters were obtained from blood samples immediately after admission to the hospital for treatment (n = 288). Statistical significance was examined using the Spearman correlation coefficient. Values of *p* < 0.05 were considered statistically significant.

Variables	Albumin (g/L)			D-dimer (ug/mL)			Vitamin D (ng/mL)		
	Spearman’s Rho	*p* Value	N	Spearman’s Rho	*p* Value	N	Spearman’s Rho	*p* Value	N
WBC (10^9^/L)	−0.332	<0.0005	285	0.261	<0.0005	284	−0.151	0.011	285
Lym (10^9^/L)	0.432	<0.0005	285	−0.418	<0.0005	284	0.034	0.562	285
PLT (10^9^/L)	−0.134	0.024	285	0.119	0.045	284	−0.110	0.063	285
BG (mmol/L)	−0.345	<0.0005	285	0.332	<0.0005	284	−0.134	0.024	285
K (mmol/L)	0.091	0.126	281	−0.045	0.454	280	−0.016	0.787	281
CRP (mg/L)	−0.651	<0.0005	286	0.626	<0.0005	285	−0.071	0.229	286
PCT (ng/mL)	−0.466	<0.0005	285	0.488	<0.0005	284	−0.087	0.141	285
AST (IU/L)	−0.341	<0.0005	286	0.429	<0.0005	285	−0.027	0.655	286
ALT (IU/L)	−0.139	0.018	286	0.232	<0.0005	285	−0.018	0.765	286
CKMB (U/L)	−0.247	<0.0005	284	0.241	<0.0005	283	−0.174	0.003	284
LDH (U/L)	−0.491	<0.0005	286	0.595	<0.0005	285	−0.096	0.107	286
pro-BNP (pg/mL)	−0.658	<0.0005	283	0.589	<0.0005	282	−0.248	<0.0005	283
Fibrinogen (g/L)	−0.488	<0.0005	264	0.501	<0.0005	265	0.067	0.276	265
PT (s)	−0.377	<0.0005	253	0.331	<0.0005	254	−0.103	0.100	255
INR	−0.342	<0.0005	257	0.206	0.001	258	−0.136	0.029	259
hsTnI (ng/mL)	−0.272	<0.0005	283	0.251	<0.0005	282	−0.107	0.072	283
Ferritin (ug/L)	−0.462	<0.0005	285	0.478	<0.0005	284	0.024	0.682	285

Spearman’s correlation coefficient. Values of *p* < 0.05 were considered statistically significant.

**Table 4:** Prediction of the probability of developing a severe clinical picture in patients with COVID-19. The serum values of the biochemical parameters were obtained from blood samples immediately after admission to the hospital for treatment (n = 288). Statistical significance was examined using the binary logistic regression. Values of *p* < 0.05 were considered statistically significant.

	B	S.E.	Wald	df	*p* Value	Exp(B)	95% C.I. for EXP(B)	
							Lower	Upper
Albumin (g/L)	−0.276	0.034	64.833	1	<0.0005	0.759	0.710	0.812
D-dimer (ug/mL)	−0.011	0.031	0.114	1	0.736	0.989	0.931	1.052
Vitamin D (ng/mL)	0.000	0.007	0.005	1	0.946	1.000	0.985	1.014
Constant	10.759	1.350	63.483	1	<0.0005	47,048.223		

**Table 5:** Prediction of the probability of developing a fatal outcome in patients with COVID-19. The serum values of the biochemical parameters were obtained from blood samples immediately after admission to the hospital for treatment (n = 288). Statistical significance was examined using the binary logistic regression. Values of *p* < 0.05 were considered statistically significant.

	B	S.E.	Wald	df	*p* Value	Exp(B)	95% C.I. for EXP(B)	
							Lower	Upper
Albumin (g/L)	−0.194	0.046	18.144	1	<0.0005	0.823	0.753	0.900
D-dimer (ug/mL)	−0.026	0.062	0.179	1	0.672	0.974	0.863	1.099
Vitamin D (ng/mL)	0.010	0.007	1.965	1	0.161	1.010	0.996	1.025
Constant	4.261	1.552	7.541	1	0.006	70.910		

## Data Availability

The datasets used and/or analyzed during the current study are available from the corresponding author upon reasonable request.
